# Prognostic significance of preoperative systemic inflammation response index in newly diagnosed glioblastoma patients underwent gross total resection: a propensity score matching analysis

**DOI:** 10.1186/s12957-022-02588-0

**Published:** 2022-04-29

**Authors:** Zhihao Wang, Junhong Li, Yunbo Yuan, Tengfei Li, Mingrong Zuo, Yanhui Liu

**Affiliations:** Department of Neurosurgery, West China Hospital of Sichuan University, Chengdu, 610041 Sichuan Province People’s Republic of China

**Keywords:** Systemic inflammation response index (SIRI), Neutrophil to lymphocyte ratio (NLR), Glioblastoma (GBM), Gross total resection (GTR), Prognosis

## Abstract

**Background:**

Glioblastoma (GBM) is the most frequent and lethal brain tumor, which possesses highly malignant characteristics and predominates in elder patients. Systemic inflammatory response index (SIRI) is a novel prognostic marker from peripheral blood, which is defined as neutrophil count × monocyte count/lymphocyte count. In the current research, we aim to explore the relationship between SIRI and newly diagnosed GBM underwent gross total resection (GTR).

**Methods:**

A retrospective analysis was conducted on consecutive newly diagnosed GBM patients underwent operation at West China Hospital from March 2015 to January 2019. X-tile software was used to determine the optimal cut-off values of SIRI, and neutrophil to lymphocyte ratio (NLR). All statistical analyses were performed using SPSS software and R software. Propensity score matching (PSM) was conducted to adjust for imbalance of all potential confounding covariates.

**Results:**

The current research included a total of 291 consecutive newly diagnosed GBM patients underwent gross total resection. Among them, 186 were male patients and 105 were female patients. In original cohort, only gender was evidently related to SIRI level. SIRI and NLR were independent prognostic indicators both in original cohort and PSM cohort. Prognostic models based on the independent prognostic factors were established, and prognostic capacity of Model SIRI was superior to Model NLR.

**Conclusion:**

In the current research, SIRI was determined to be an independent prognostic indicator for GBM. And the prognostic predictive ability of SIRI was stronger than NLR.

**Supplementary Information:**

The online version contains supplementary material available at 10.1186/s12957-022-02588-0.

## Introduction

Glioblastoma (GBM) is the most common and lethal primary brain tumor, which is highly invasive but not metastatic, namely, it is confined to the central nervous system [1]. The median survival is 15 months in GBM patients who have though accepted aggressive combination of therapies including maximal safe resection, adjuvant radiotherapy, and adjuvant temozolomide (TMZ) treatment [2]. This incurable malignancy brings heavy financial burden on health care system all over the world. Surgery dominates various therapeutic schemes. Compared with subtotal resection (STR) and biopsy, gross total resection (GTR) is proved to effectively prolong overall survival (OS) and progression-free survival (PFS) [3, 4].

Inflammation is deemed as a hallmark of cancer development and progression, which is essential for tumor growth, invasion, angiogenesis, and metastasis [5, 6]. Systemic inflammation varies in development with tumor type and stage, and it remains unclear about the complicated mechanisms of systemic inflammatory response in in cancer patients [7]. Lots of peripheral blood-related inflammatory markers have been applied in clinical practice to determine and quantify the systemic inflammatory response. The representative markers include neutrophil to lymphocyte ratio (NLR), derived NLR (dNLR), lymphocyte to monocyte ratio (LMR), platelet to lymphocyte ratio (PLR), and Glasgow prognostic score (GPS). These markers are confirmed to have strong prognostic and predictive abilities in various tumors [7–13]. Compared with the above-mentioned markers, systemic inflammatory response index (SIRI) is a novel prognostic marker. And SIRI is reported to play a significant prognostic role in cancers like cervical cancer, head and neck squamous cell carcinoma, breast cancer, and gallbladder cancer [14–18].

Malignant progression of glioma is also connected with systemic inflammatory response. Preoperative hematological markers levels vary among glioma grades and have predictive ability [19]. Although the pathophysiological mechanisms of systemic inflammatory response of glioma also remains unknown, there is a hypothesis that it is generated when local inflammatory cells leak into the circulatory system from broken blood-brain barrier. As regard to GBM, it is reported that the markers including NLR, PLR, systemic immune-inflammation (SII), and prognostic nutrition index (PNI) have strong prognostic abilities in specific patient populations [20–23]. In the current research, we aim to explore the relationship between SIRI and newly diagnosed GBM with GTR. And with propensity score matching, we will further evaluate the prognostic value of SIRI in GBM patients.

## Materials and methods

### Patients

This was a retrospective analysis on consecutive newly diagnosed GBM patients who had underwent operation at West China Hospital from March 2015 to January 2019. All these patients underwent a craniotomy on GBM with GTR, and their baseline clinical data were retrieved from the electronic medical record system. The extent of resection was determined by surgical records and postoperative imaging including MRI and CT within 72 h after surgery. The pathological diagnosis criteria were followed the 2016 WHO classification of CNS tumors, and two independent pathologists verified the diagnoses of tumor specimens before 2017. These patients were followed up until January 2021.

The exclusion criteria were (1) younger than 18-year-old; (2) not GTR; (3) absence of definite pathological diagnosis; (4) incomplete baseline clinical data; (5) absence of preoperative magnetic resonance imaging (MRI); (6) receiving adjuvant therapy before operation; (7) receiving corticosteroids therapy before admission; (8) presence of history of infectious diseases or blood system diseases or in a low nutrition condition before surgery; (9) recurrent; (10) lost to follow-up at any stage of the disease.

### Parameters assessment

The following clinical variables were retrieved from electronic medical record system: gender, age at diagnosis, preoperative Karnofsky performance status (KPS) score, presence of preoperative seizures, tumor locations, Ki-67 index, status of isocitrate dehydrogenase 1 (IDH-1), conditions of adjuvant therapy, and blood test results. Routine blood test was conducted within 1 week before operation. SIRI was defined as neutrophil count × monocyte count/lymphocyte count, and NLR was defined as neutrophil count/lymphocyte count.

After initial treatment, the patients were followed up every 3 months in the first year, and every 6 months thereafter. OS was defined as the duration from the date of operation to death or the end of the observation period.

### Statistical analysis

X-tile software was used to determine the optimal cut-off values of SIRI and NLR [24].

All statistical analyses were performed using SPSS software (Version 22.0, IBM Co., Armonk, NY, USA) and R software (Version 3.6.1). Continuous variables were presented as mean ± standard deviation (SD), and categorical variables were presented as frequency and percentage. Data that conformed to the normal distribution was compared using Student’s *t* test, otherwise Mann-Whitney *U* test was applied. Three or more independent groups were compared by using one-way analysis of variance (ANOVA) or Kruskal-Wallis test. Kaplan-Meier (K-M) curves were applied to calculate cumulative OS using the log-rank test. The Cox regression analysis were employed to determine the influences of risk factors for overall survival in GBM patients. Univariate Cox regression was firstly conducted to evaluate clinical variables, then variables with *p* value< 0.1 were included into backward stepwise multivariate Cox regression. Harrell’s concordance index (C-index) and Akaike information criterion (AIC) were calculated to evaluate different prognostic models. A higher C-index and a lower AIC indicated better predictive performance [25]. A two-sided *p* value < 0.05 referred as statistically significant difference.

Propensity score matching was conducted to adjust for imbalance of all potential confounding covariates: gender, age at diagnosis, KPS, presence of preoperative seizures, tumor locations, Ki-67 index, IDH-1 mutation status, and conditions of adjuvant therapy. These patients were matched 1:1 using the nearest-neighbor algorithm with a caliper width of 0.2 and without replacement.

## Results

### Baseline characteristics

**Fig. 1:**
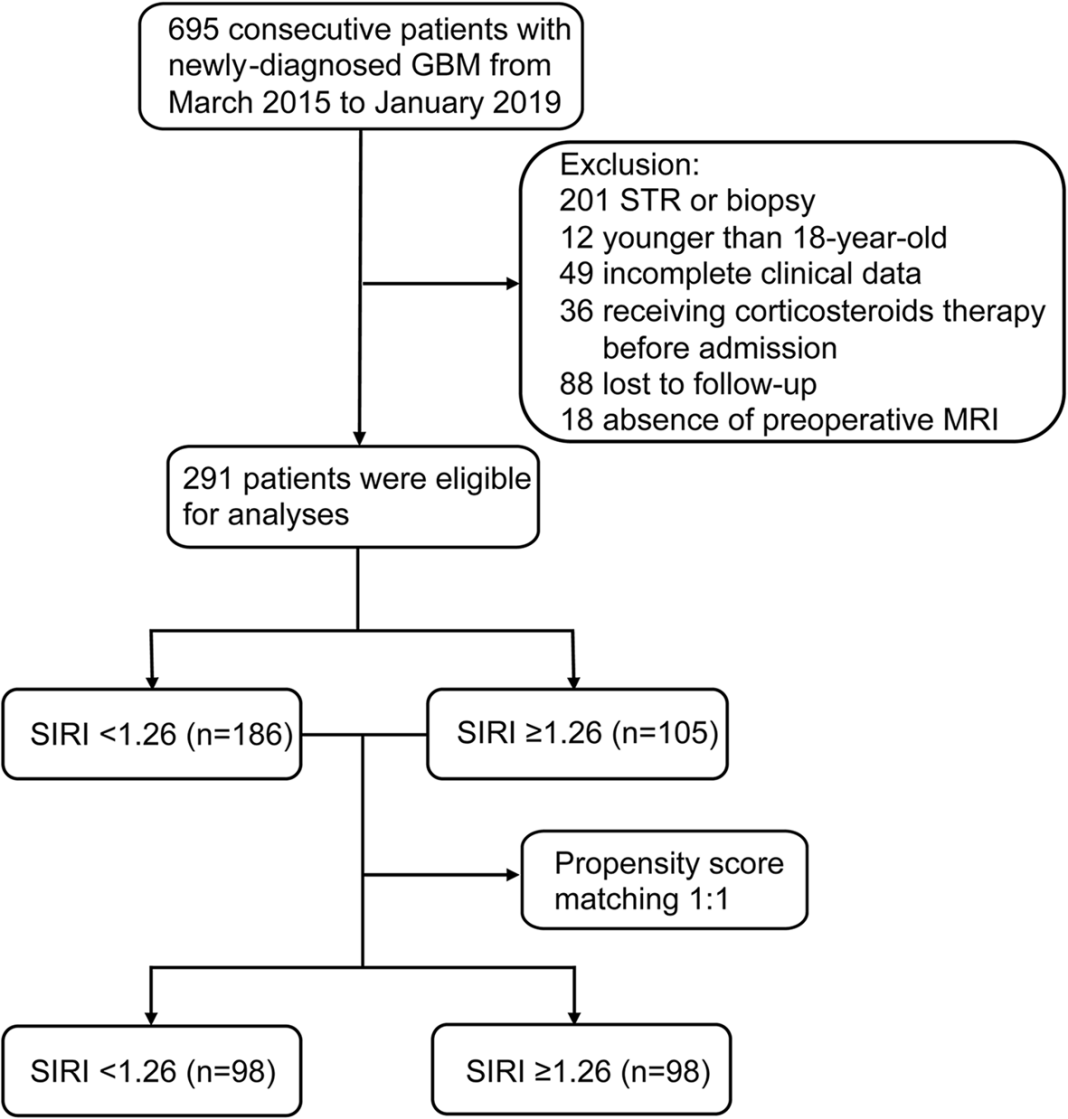
Flow chart of the current study. Abbreviations: GBM, glioblastoma; STR, subtotal resection; MRI, magnetic resonance imaging; SIRI, systemic inflammation response index

After screening (Fig. 1), a total of 291 consecutive newly diagnosed GBM patients including 186 men and 105 women underwent gross total resection were included in the current research. (Table 1) The mean and median OS were 435.6 days and 355 days respectively, ranging from 31 to 1580 days. The mean and median age at diagnosis in the cohort was 53.7 and 54 years respectively. Among them, 40 patients had preoperative seizures, and 108 patients had better KPS score (> 80). The number of tumors in left and right hemispheres was roughly the same (144 vs 132), while 15 cases had tumors in midline area or involving both sides of the brain. As regard to tumor location, 98 foci were located at frontal lobes, 59 at temporal lobes, 26 at parietal lobes, 8 at occipital lobes, 12 at insular lobes, while 88 GBMs involved multiple lobes, structures, or cerebellum. A total of 230 patients received chemotherapy and radiotherapy, and most of the treatments were performed according to the Stupp’s regimen which contained 42–day concomitant radiochemotherapy and subsequent 6–12 consecutive cycles of temozolomide alone. The other 61 patients did not receive chemotherapy or radiotherapy or abandoned treatment at an early stage due to the deteriorative physical condition or severe side effects. Two pathological biomarkers were included in clinical variables. Ki-67 index greater than or equal to 30% was observed in 121 GBMS, and IDH-1 mutation was found in 42 patients.

**Table 1:** Baseline clinical characteristics of glioblastoma patients in original and PSM cohort

Clinical characteristic		Original cohort	PSM cohort
**Sample size**		291 (100)	196 (100)
**Overall survival**	Mean ± SD (day)	435.6 ± 314.0	389.2 ± 282.8
	Median (range)	355 (31–1580)	304 (31–1580)
**Age at diagnosis**	Mean ± SD (year)	53.7 ± 14.2	54.2 ± 13.8
	Median (range)	54 (18–85)	55 (19–85)
**Gender**	Male	186 (63.9)	147 (75.0)
	Female	105 (36.1)	49 (25.0)
**Preoperative seizures**	Yes	40 (13.7)	28 (14.3)
	No	251 (86.3)	168 (85.7)
**Karnofsky performance status**	≤ 80	183 (62.9)	123 (62.8)
	> 80	108 (37.1)	73 (37.2)
**Hemisphere**	Right	132 (45.4)	89 (45.4)
	Left	144 (49.5)	98 (50.0)
	Midline or bilateral	15 (5.1)	9 (4.6)
**Location**	Frontal lobe	98 (33.7)	60 (30.6)
	Temporal lobe	59 (20.3)	43 (21.9)
	Parietal lobe	26 (8.9)	18 (9.2)
	Occipital lobe	8 (2.7)	3 (1.5)
	Insular lobe	12 (4.1)	10 (5.1)
	Other locations	88 (30.2)	62 (31.6)
**Adjuvant therapy**	Yes	230 (79.0)	156 (79.6)
	No or undone	61 (23.0)	40 (20.4)
**Ki-67**	< 30%	170 (58.4)	119 (60.7)
	≥ 30%	121 (41.6)	77 (39.3)
**IDH-1**	Mutant	42 (14.4)	27 (13.8)
	Wildtype	249 (85.6)	169 (86.2)
**SIRI**	< 1.26	177 (60.8)	98 (50.0)
	≥ 1.26	114 (39.2)	98 (50.0)
**NLR**	< 4.86	237 (81.4)	–
	≥ 4.86	54 (18.6)	–
	< 4.63	–	146 (74.5)
	≥ 4.63	–	50 (25.5)

Data are presented as *n* (%)

*Abbreviations*: *IDH-1* isocitrate dehydrogenase-1, *SIRI* systemic inflammation response index, *NLR* neutrophil to lymphocyte ratio, *PSM* propensity score matching

The optimal cut-off points of SIRI and NLR were 1.26 and 4.86 respectively (Supplementary Figure 1). In the original cohort, 177 patients showed SIRI < 1.26 while 114 with SIRI ≥ 1.26, and 237 patients had NLR < 4.86 while NLR≥4.86 was found in 54 patients.

To reveal the potential confounding bias between patients with SIRI level < 1.26 and ≥ 1.26, PSM was performed and a new cohort including 196 patients was available for further analyses. Clinical characteristics of patients in PSM cohort were listed in Table 1. Of note, after PSM, the optimal cut-off value of NLR was changed to 4.63 (Supplementary Figure 1). NLR < 4.63 and NLR ≥ 4.63 were found in 146 and 50 patients respectively.

### Association between SIRI and clinical variables

As shown in Table 2, in original cohort, only gender was evidently related to SIRI level (*p* < 0.001), which indicated that male patients tended to have higher SIRI. In PSM cohort, there was not any clinical variable significantly associated with SIRI.

**Table 2:** Relationship between SIRI and clinical variables

Clinical variables		Original cohort			PSM cohort		
		SIRI < 1.26 (*n* = 177)	SIRI ≥ 1.26 (*n* = 114)	*p* value	SIRI < 1.26 (*n* = 98)	SIRI ≥ 1.26 (*n* = 98)	*p* value
Age at diagnosis	< 55	94	55	0.933	50	46	0.988
	≥ 55	83	59	–	48	52	–
Gender	Male	96	90	***< 0.001***	73	74	0.177
	Female	81	24	–	25	24	–
Preoperative seizure	Yes	25	15	0.83	15	13	0.974
	No	152	99	–	83	85	–
KPS	≤ 80	103	80	0.396	59	64	0.959
	> 80	74	34	–	39	34	–
Hemisphere	Right	74	58	0.11	43	46	0.465
	Left	94	50		51	47	–
	Midline or bilateral	9	6	–	4	5	–
Tumor location	Frontal lobe	70	28	0.088	32	28	0.136
	Temporal lobe	29	30	–	16	27	–
	Parietal lobe	20	6	–	12	6	–
	Occipital lobe	6	2	–	1	2	–
	Insular lobe	7	5	–	5	5	–
	Other regions	45	43	–	32	30	–
Ki-67 index	< 30%	105	65	0.636	63	56	0.217
	≥ 30%	72	49	–	35	42	–
IDH-1 status	Mutant	29	13	0.531	15	12	0.491
	Wildtype	148	101	–	83	86	–

Significant findings are expressed in bold and italic

*Abbreviations*: *KPS* Karnofsky performance status, *IDH-1* isocitrate dehydrogenase-1, *SIRI* systemic inflammation response index, *NLR* neutrophil to lymphocyte ratio, *PSM* propensity score matching

### Prognostic value of SIRI

**Fig. 2:**
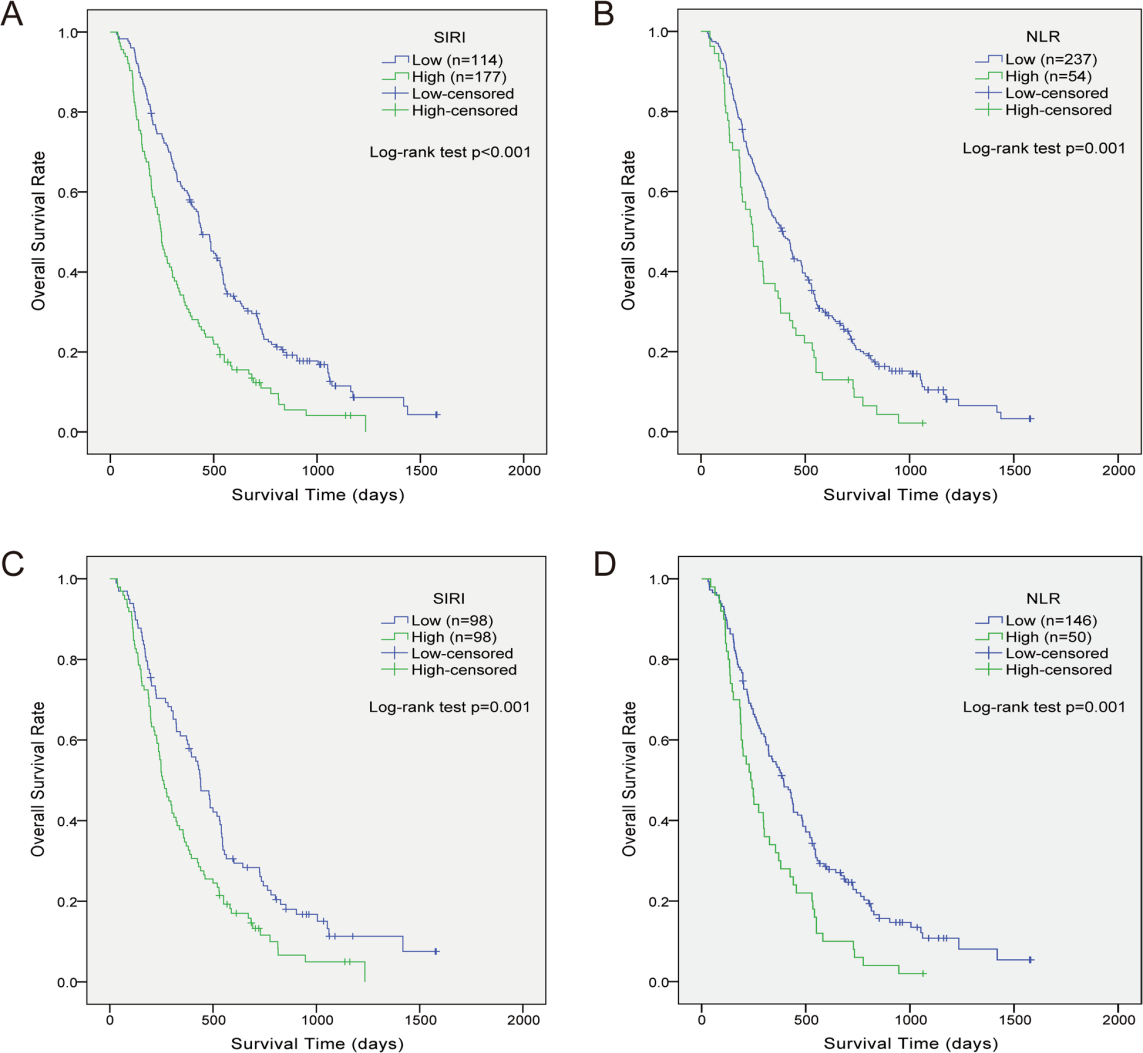
Kaplan-Meier curves showing overall survival of GBM patients stratified by value of SIRI (**A**, **C**) and NLR (**B**, **D**) in original and PSM cohort respectively. Abbreviations: SIRI, systemic inflammation response index; NLR, neutrophil to lymphocyte ratio; PSM, propensity score matching

K-M curves indicated the reliability of optimal cut-off points (Fig. 2). In univariate Cox regression, age at diagnosis, gender, KPS, adjuvant therapy, IDH-1 status, NLR, and SIRI were considered to affect OS (*p* < 0.05) in original cohort (Table 3). After multivariate analysis, age (HR1.314, 95% CI 1.011–1.707, *p* = 0.041), gender (HR 0.699, 95% CI 0.528-0.925, *p* = 0.012), adjuvant therapy (HR 2.151, 95%CI 1.587–2.916, *p* < 0.001), Ki-67 index (HR 1.316, 95%CI 1.019–1.699, *p* = 0.036), IDH-1 status (HR 2.384, 95% CI 1.560–3.643, *p* < 0.001), SIRI (HR 1.646, 95%CI 1.253–2.163, *p* < 0.001), and NLR (HR 1.392, 95% CI 1.013–1.912, *p* = 0.041) were confirmed as independent predictors for OS.

**Table 3:** Univariate and multivariate Cox regression for risk factors predictive of GBM in original cohort

Clinical variables		Univariate analysis			Multivariate analysis		
		HR	95% CI	*p*	HR	95% CI	*p*
**Age at diagnosis**	< 55	Reference	–	–	Reference	–	–
	≥ 55	1.699	1.325–2.180	***< 0.001***	1.314	1.011–1.707	***0.041***
**Gender**	Male	Reference	–	–	Reference	–	–
	Female	0.626	0.482–0.814	***< 0.001***	0.699	0.528–0.925	***0.012***
**KPS**	≤ 80	Reference	–	–	Reference	–	–
	> 80	0.741	0.573–0.958	***0.022***	0.874	0.670–1.141	0.322
**Hemisphere**	Right	Reference	–	–	–	–	–
	Left	0.826	0.641–1.064	0.139	–	–	***–***
	Midline or bilateral	1.478	0.848–2.575	0.168	–	–	–
	Frontal lobe	Reference	–	–	–	–	–
	Temporal lobe	1.314	0.935–1.847	0.115	–	–	–
**Location**	Parietal lobe	0.893	0.553–1.443	0.645	–	–	–
	Occipital lobe	0.923	0.426–2.002	0.84	–	–	–
	Insular lobe	0.929	0.466–1.850	0.834	–	–	–
	Other regions	1.632	1.198–2.222	***0.002***	–	–	–
**Preoperative seizures**	No	Reference	–	–	–	–	–
	Yes	1.231	0.868–1.747	0.244	–	–	–
**Adjuvant therapy**	Yes	Reference	–	***–***	Reference	–	***–***
	No or undone	2.059	1.533–2.764	***< 0.001***	2.151	1.587–2.916	***< 0.001***
**Ki67**	< 30%	Reference	–	–	Reference	–	–
	≥30%	1.261	0.983–1.618	0.068	1.316	1.019–1.699	***0.036***
**IDH-1**	Mutant	Reference	–	–	Reference	–	–
	Wildtype	2.867	1.917–4.288	***< 0.001***	2.384	1.560–3.643	***< 0.001***
**SIRI**	Low	Reference	–	–	Reference	–	–
	High	1.843	1.431-2.373	***< 0.001***	1.646	1.253–2.163	***< 0.001***
**NLR**	Low	Reference	–	***–***	Reference	–	–
	High	1.67	1.228-2.270	***0.001***	1.392	1.013–1.912	***0.041***

Significant findings are expressed in bold and italic

*Abbreviations*: *HR* hazard ratio, *CI* confidence interval, *KPS* Karnofsky performance status, *IDH-1* isocitrate dehydrogenase-1, *SIRI* systemic inflammation response index, *NLR* neutrophil to lymphocyte ratio, *PSM* propensity score matching

As shown in Table 4, in PSM cohort, similarly, SIRI (HR 1.641, 95% CI 1.206–2.234, *p* = 0.002) and NLR (HR 1.570, 95% CI 1.108–2.226, *p* = 0.011) were independent prognostic indicators based on the results of multivariate Cox regression. Other independent indicators included age at diagnosis, condition of adjuvant therapy, and IDH-1 status.

**Table 4:** Univariate and multivariate Cox regression for risk factors predictive of GBM in PSM cohort

Clinical variables		Univariate analysis			Multivariate analysis		
		HR	95% CI	*p*	HR	95% CI	–
**Age at diagnosis**	< 55	Reference	–	–	Reference	–	–
	≥ 55	1.976	1.447–2.697	***< 0.001***	1.441	1.042–1.993	***0.027***
**Gender**	Male	Reference	–	–	Reference	–	–
	Female	0.613	0.429–0.875	***0.007***	0.758	0.521–1.101	0.146
**KPS**	≤ 80	Reference	–	–	Reference	–	–
	> 80	0.745	0.543–1.022	0.068	0.83	0.598-1.153	0.267
**Hemisphere**	Right	Reference	–	–	–	–	–
	Left	0.982	0.724–1.332	0.906	–	–	–
	Midline or bilateral	1.772	0.817–3.846	0.148	–	–	–
	Frontal lobe	Reference	–	–	–	–	–
	Temporal lobe	1.407	0.930–2.130	0.106	–	–	–
**Location**	Parietal lobe	0.892	0.502–1.586	0.698	–	–	–
	Occipital lobe	0.870	0.314–2.416	0.790	–	–	–
	Insular lobe	0.734	0.333–1.620	0.444	–	–	–
	Other regions	1.353	0.924–1.980	0.121	–	–	–
**Preoperative seizures**	No	Reference	–	–	–	–	–
	Yes	1.136	0.725–1.780	0.578	–	–	–
**Adjuvant therapy**	Yes	Reference	–	–	Reference	–	–
	No or undone	1.751	1.211–2.533	***0.003***	1.685	1.158-2.451	***0.006***
**Ki67**	< 30%	Reference	–	–	–	–	–
	≥ 30%	1.206	0.890–1.635	0.227	–	–	–
**IDH-1**	Mutant	Reference	–	–	Reference	–	–
	Wildtype	3.327	1.989–5.566	***< 0.001***	2.958	–	***< 0.001***
**SIRI**	Low	Reference	–	–	Reference	–	–
	High	1.637	1.209–2.217	***0.001***	1.641	–	***0.002***
**NLR**	Low	Reference	–	–	Reference	–	–
	High	1.751	1.253–2.447	***0.001***	1.57	1.108–2.226	***0.011***

Significant findings are expressed in bold and italic

*Abbreviations*: *HR* hazard ratio, *CI* confidence interval, *KPS* Karnofsky performance status, *IDH-1* isocitrate dehydrogenase-1, *SIRI* systemic inflammation response index, *NLR* neutrophil to lymphocyte ratio, *PSM* propensity score matching

### Prognostic model based on SIRI and NLR

To compare the prognostic ability of SIRI and NLR in the current two cohorts, prognostic models were established based on the independent prognostic factors (Table 5). In original cohort, in addition to SIRI and NLR, other 5 variables including age at diagnosis, gender, adjuvant therapy, Ki-67 index, and IDH-1 status were involved in the composition of the models. Model SIRI had higher C-index and lower AIC compared with Model NLR (C-index 0.672 VS 0.659, AIC 2410.09 VS 2418.68). In PSM cohort, each model had 4 variables. As the same, prognostic ability of Model SIRI was superior to that of Model NLR (C-index 0.656 VS 0.650, AIC 1516.63 VS 1518.11).

**Table 5:** Prognostic model based on SIRI and NLR for GBM patients

Clinical variables		Prognostic models											
		Original cohort (model SIRI)			Original cohort (model NLR)			PSM cohort (model SIRI)			PSM cohort (model NLR)		
		HR	95% CI	*p* value	HR	95% CI	*p* value	HR	95% CI	*p* value	HR	95% CI	*p* value
**Age at diagnosis**	< 55	Reference	–	–	Reference	–	–	Reference	–	–	Reference	–	–
	≥ 55	1.314	1.011–1.707	***0.041***	1.340	1.032–1.740	***0.028***	1.441	1.042–1.993	***0.027***	1.516	1.099–2.090	***0.011***
**Gender**	Male	Reference	–	–	Reference	–	–	–	–	–	–	–	–
	Female	0.699	0.528–0.925	***0.012***	0.618	0.473–0.807	***< 0.001***	–	–	–	–	–	–
**Adjuvant therapy**	Yes	Reference	–	–	Reference	–	–	Reference	–	–	Reference	–	–
	No or undone	2.151	1.587–2.916	***< 0.001***	2.139	1.569–2.914	***< 0.001***	1.685	1.158–2.451	***0.006***	1.511	1.030–2.216	***0.035***
**Ki67**	< 30%	Reference	–	–	Reference	–	–	–	–	–	–	–	–
	≥ 30%	1.316	1.019–1.699	***0.036***	1.274	0.988–1.644	0.062	–	–	–	–	–	–
**IDH-1**	Mutant	Reference	–	–	Reference	–	–	Reference	–	–	Reference	–	–
	Wildtype	2.384	1.560–3.643	***< 0.001***	2.404	1.575–3.668	***< 0.001***	2.958	1.729–5.063	***< 0.001***	2.837	1.658–4.855	***< 0.001***
**SIRI**	Low	Reference	–	–	–	–	–	Reference	–	–	–	–	–
	High	1.646	1.253-2.163	***< 0.001***	–	–	–	1.641	1.206–2.234	***0.002***	–	–	–
**NLR**	Low	–	–	–	Reference	–	–	–	–	–	Reference	–	–
	High	–	–	–	1.392	1.013–1.912	***0.041***	–	–	–	1.601	1.132–2.265	***0.008***
**C-index**		0.672			0.659			0.656			0.650		
**AIC**		2410.09			2418.68			1516.63			1518.11		

Significant findings are expressed in bold and italic

*Abbreviations*: *HR* hazard ratio, *CI* confidence interval, *IDH-1* isocitrate dehydrogenase-1, *SIRI* systemic inflammation response index, *NLR* neutrophil to lymphocyte ratio, *PSM* propensity score matching, *C-index* Harrell’s concordance index, *AIC* Akaike information criterion

## Discussion

As grade 4 glioma, GBM possesses highly malignant characteristics and predominates in patients over 55 years of age [26]. Through the years, oncology researchers have stressed on less invasive and convenient methods to detect and monitor tumor growth, progression, and even real-time treatment response. Peripheral blood markers and liquid biopsy markers including circulating tumor cells, circulating tumor DNA, extracellular vesicles, and exosomes certainly have the potential to change the dynamics of cancer management and treatment [27, 28]. In the current research, we attempted to explore the relationship between pretreatment peripheral blood SIRI and newly diagnosed GBM with GTR. We found SIRI can serve as an independent prognosis indicator for newly diagnosed GBM after GTR. Though NLR had similar independent prognostic ability in same cohort, SIRI seemed to have better predictive ability on prognosis.

Hematological markers are of intense interest in current clinical cancer research [29, 30]. These markers are accessible and the tests are mostly harmless so that large-scale prospective and retrospective studies have been conducted. Tumor microenvironment is associated to cascades of inflammation such as platelet activation, stimulation of coagulation, and subsequent release of inflammatory cytokines [6]. Transcription factors, cytokines, chemokines, and infiltrating leukocytes are key orchestrators of the inflammation-mediated tumor progression [31]. Local immune response leads to changes of systemic inflammation, therefore peripheral blood markers are theoretically indirectly connected to tumor progression. In fact, large trials also verify that cancer-related inflammatory markers have prognostic relevance and even interact with adjuvant therapy [32–34].

It is still unclear about the underline mechanism of the relationship between systemic inflammation and tumor prognosis. And it also remains unknown about the specific role of immune cells that are adjacent to or infiltrate into the tumor due to the heterogeneous functions of inflammatory cells that may promote tumor progression, and alternatively may lead to tumor cell destruction [35]. At the same time, tumor-related systemic and local inflammatory responses are considered to be crucial therapeutic targets for cancer treatment. Targeting inflammatory mediators like immune cells, stromal cells, fibroblasts, and endothelial cells might be effective in controlling the inflammatory response in cancer patients [36]. It is an issue about how to tip the balance between cancer-promoting inflammatory responses and cancer-inhibiting inflammatory responses [37].

NLR, the ratio of systemic neutrophils to lymphocytes, has prognostic value in various tumors. In the field of glioma, a meta-analysis included 16 studies from Lei et al. indicated that high NLR was considered a high-risk prognostic factor in gliomas [38]. SIRI, a modified version of NLR, is highly similar to NLR in structure. In previous studies, the optimal cut-off values of pretreatment SIRI ranged from 0.54 to 2.3. In our study, the optimal cut-off value calculated by X-tile software was 1.26, which was within an acceptable range. Several cancer-related studies compared the prognostic ability between SIRI and NLR in the same cohort by using receiver operating characteristic (ROC) curves, and SIRI was found to have stronger predictive capacity than NLR [39–41]. In the current study, we compared the prognostic ability between SIRI and NLR by building prognosis predictive models, which were more precise and reasonable. After adjusting potential confounders by PSM, we got the same results in the new cohort.

There were still some limitations in the current research. Firstly, other important tumor-related biomarkers like O6-methylguanine-DNA methyltransferase (MGMT) and telomerase reverse transcriptase (TERT) status that were related to prognosis were not included in the study due to insufficient pathological data. Secondly, there was not enough participants to divide the original cohort into training group and validation group, which can make results more convincing. Thirdly, there lacked of effective and practical methods to monitor the progression of GBMs after surgery due to the rapid recurrence and high early mortality. Therefore, we did not acquire precise clinical data to calculate progression-free survival, and OS was the only outcome. Fourthly, it is hard for us to regularly collect and analyze the blood from GBM patients at follow-up period, so we cannot proceed further analyses for the relationship between postoperative SIRI and GBM.

## Conclusion

To our knowledge, this is the first study focusing on prognostic significance of preoperative SIRI in newly diagnosed GBM patients underwent GTR. In the current research, SIRI was determined to be an independent prognostic marker for overall survival in GBM. And the prognostic predictive capacity of SIRI was stronger than NLR. In the future, systemic inflammation-based prognostic markers could possibly not only identify cancer patients at risk but may also potentially provide precise therapeutic targets for clinical treatment.

## Supplementary Information

**Additional file 1: Supplementary Figure 1**. X-tile software was used to calculate the optimal cut-off values of SIRI in original cohort (A) and NLR in both original (B) and PSM cohort (C). Abbreviations: SIRI, systemic inflammation response index; NLR, neutrophil to lymphocyte ratio; PSM, propensity score matching.

## Data Availability

The datasets for this study are available from the corresponding author on reasonable request.

## Abbreviations

AIC: Akaike information criterion
ANOVA: Analysis of variance
C-index: Harrell’s concordance index
CI: Confidence interval
GBM: Glioblastoma
GTR: Gross total resection
HR: Hazard ratio
IDH-1: Isocitrate dehydrogenase 1
K-M: Kaplan-Meier
KPS: Karnofsky performance status
LMR: Lymphocyte to monocyte ratio
MGMT: O6-methylguanine-DNA methyltransferase
MRI: Magnetic resonance imaging
NLR: Neutrophil to lymphocyte ratio
OS: Overall survival
PSM: Propensity score matching
PFS: Progression-free survival
PLR: Platelet to lymphocyte ratio
PNI: Prognostic nutrition index
ROC: Receiver operating characteristic
SD: Standard deviation
SII: Systemic immune-inflammation
SIRI: Systemic inflammatory response index
TMZ: Temozolomide
TERT: Telomerase reverse transcriptase
