# Supplementary figures and images for: Prognostic significance of preoperative systemic inflammation response index in newly diagnosed glioblastoma patients underwent gross total resection: a propensity score matching analysis
Source: World J Surg Oncol. 2022 Apr 29;20:137. doi: 10.1186/s12957-022-02588-0 (PMC9052476; doi:10.1186/s12957-022-02588-0)

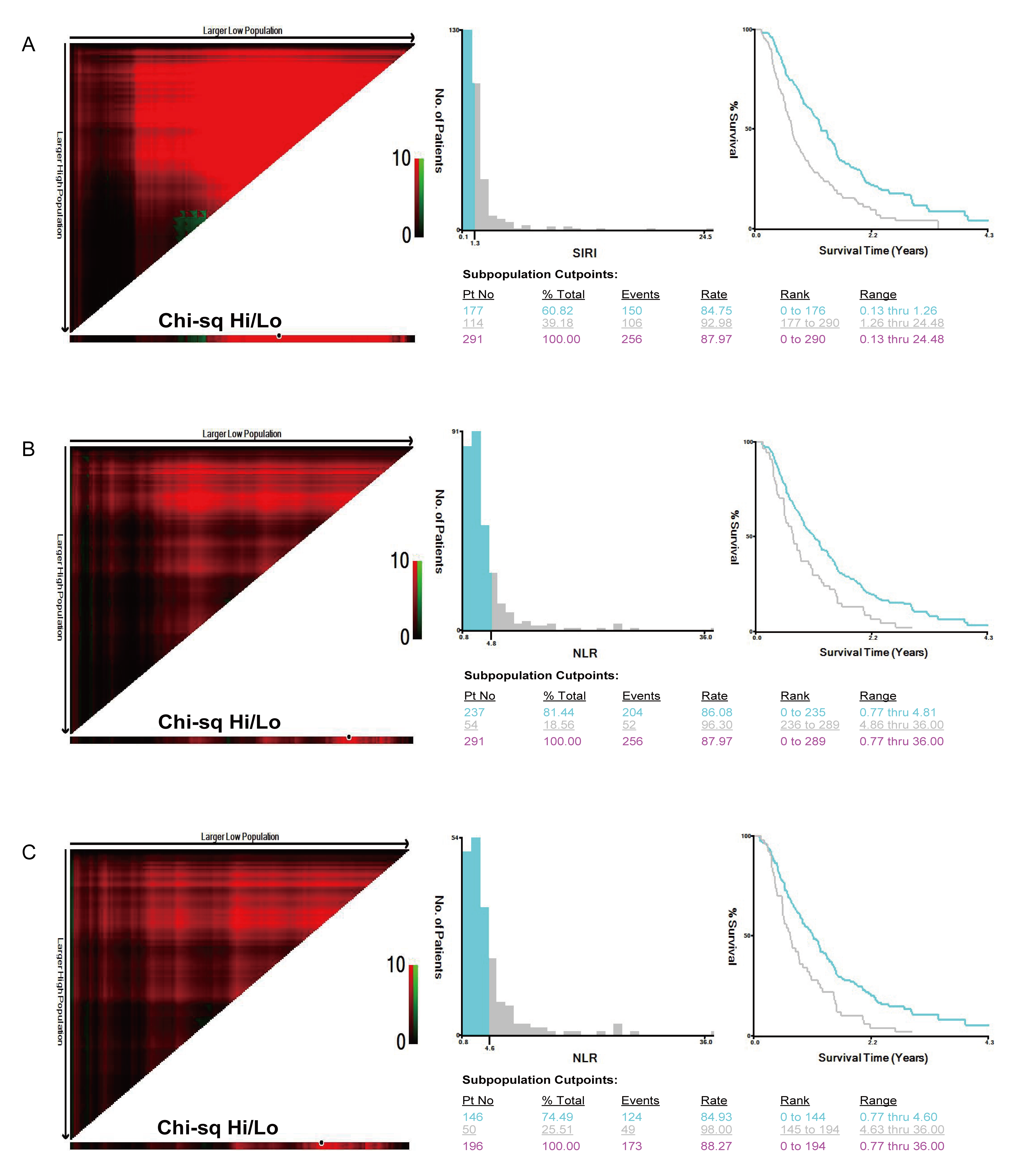

Supplement: Supplementary file 1 — Additional file 1: Supplementary Figure 1. X-tile software was used to calculate the optimal cut-off values of SIRI in original cohort (A) and NLR in both original (B) and PSM cohort (C). Abbreviations: SIRI, systemic inflammation response index; NLR, neutrophil to lymphocyte ratio; PSM, propensity score matching. [file 12957_2022_2588_MOESM1_ESM.tif]
